# Exploring rugby coaches perception and implementation of performance analytics

**DOI:** 10.1371/journal.pone.0280799

**Published:** 2023-01-24

**Authors:** Mikaela J. Callinan, Jonathan D. Connor, Wade H. Sinclair, Anthony S. Leicht

**Affiliations:** 1 Sport and Exercise Science, College of Healthcare Sciences, James Cook University, Townsville, Australia; 2 Rugby Australia Limited, Sydney, Australia; 3 Australian Institute of Tropical Health & Medicine, James Cook University, Townsville, Queensland, Australia; Mugla Sitki Kocman University: Mugla Sitki Kocman Universitesi, TURKEY

## Abstract

Professional coaches commonly rely on performance analysis and metrics to help make decisions regarding their practices, selection and tactics. However, few studies to date have explored coaches’ perspectives of performance analysts successful integration into the high-performance environment. The aim of this study was to investigate coaches’ philosophies surrounding performance analysis and how they perceived analysts could support and implement these approaches into coaching practices and match preparation. Semi-structured interviews were conducted with five professional elite level Rugby Union coaches to investigate their perceptions of performance analysis, and the contribution of performance analysts to the high-performance environment. Results revealed three main dimensions, including the role, purpose, and desired attributes of a performance analyst. Firstly, the role of the analyst was described in terms of being an *information specialist*, who collects, filters, and delivers information to stakeholders, and a *generalist*, who helps coaches utilise technology. Secondly, the purpose of the analyst was described in terms of providing both accountability and support for coaches and players. Finally, the attributes needed of an analyst included the ability to form a close relationship with coaches, communicate complex information in meaningful ways, and who was proactive, innovative, and creative when tasked with delivering information. The findings highlighted the crucial roles, purposes, and attributes of a performance analyst within high-performance Rugby Union identified by coaches and the importance of the coach-analyst relationship to support these dimensions.

## Introduction

Performance analysis (PA) has become increasingly popular and accessible within professional sport [1] with professional coaches relying upon various sources of PA information and results for their short-, medium- and long-term planning throughout the season [2]. Within the professional sporting environment, metrics such as the number of accelerations and maximum velocity have been obtained via wearable microtechnology (e.g. Global/Local Positioning Systems, Global Navigation Satellite Systems) as well as metrics such as number of successful linebreaks, total number of turnovers, and the number of tackles made or percentage of tackle success through manual or automated notational analysis (e.g. Opta Sports™ and StatsPerform™). Alongside the collection of metrics, PA also utilises video footage to further support the quantitative data as well as providing coaches and athletes with visual feedback to change behaviours and further develop understanding and awareness [3,4]. With increased multimodal technology, there has been an abrupt concomitant rise in available PA for coaches to influence their practices, selections, and tactics [2,5,6]. In response, performance analysts have been employed to collate and filter ‘big data’ into meaningful information for coaches and players. Although several studies have explored which metrics may partially predict successful performance [7–10], there is currently a lack of empirical understanding of how coaches and analysts interact in the high-performance environment to reduce big rugby data to key metrics.

Across various sports, PA has been used by coaches to improve athletic performance [11] and strategically support technical and tactical decisions surrounding team and/or individual performances [12]. Previously, Wright, Atkins [2] examined elite coaches’ engagement, involvement and implementation of PA processes across many team sports including rugby league, hockey, football, basketball and rugby union (RU). These authors identified that coaches regularly utilised PA tools to provide athlete feedback in preparation for upcoming opposition, gather quantitative match data, and inform their decision-making and planning of training sessions [2]. Additionally, coaches often integrated PA with athletes’ preparatory activities (e.g., reviewing video footage) to increase their confidence levels for upcoming matches and improve athlete understanding and development [13,14]. Subsequently, the successful delivery of PA information during previews of upcoming matches, or reviews of past match performances has become increasingly important to influence match tactics, athlete preparation and overall athletic performance outcomes [15].

Although PA has been used regularly in a range of sports [1,5,7,16], minimal empirical research has examined the connection between PA and coaching application, particularly in regards to RU [11]. Rugby Union is a high-profile international sport that involves multiple stakeholders including coaches, athletes, and other high performance support staff that are directly involved in performance outcomes. While these stakeholders are key to team success, they may have variable, or possibly limited, knowledge of important PA metrics and processes. Furthermore, a coach may not necessarily know what information is most critical to successful performance, or how to successfully apply PA into their coaching practices. Therefore, coaches rely on the performance analyst to collect, interpret, and effectively deliver information to help inform their decision-making for successful team performances [17].

The interaction of PA and coaches is crucial to successful RU performance outcomes [2,18] however, it’s utilisation in a high-performance RU setting [19], as well as influencing coaching processes [16], remains unclear. However, performance analysts clearly play an important role within the high-performance setting and contribute to the coaching process implemented within a team. This lack of insight into the PA-coaching nexus may be due to the secretive nature of elite sport with coaches and teams concerned that they may lose their “competitive edge” [16]. Performance analysts play an integral role within the performance operation of elite sporting teams and organisations [17,20,21] and it is crucial to understand how coaches integrate the performance analyst’s skill set into the high-performance environment. Therefore, the aim of this study was to explore elite RU coaches philosophies regarding the need for, and contributions of, performance analysts within an elite, high-performance RU environment.

## Methods

### Participants

Professional coaches (n = 5) working with elite Australian RU teams within the last 2 years, volunteered to participate in the study. All coaches were male and had an average of 13 years’ experience (SD = 5.4, Range = 5–18 years) working with semi-professional and professional RU teams that competed internationally, within the domestic competitions. All coaches had access to and experience with professional analysts (irrespective of men’s or women’s game) and worked in clubs with at least one PA, while 2 coaches have worked in environments with multiple analysts at a time. All participants provided written informed consent prior to participation with ethical approval for the study obtained from the Institutional Human Research Ethics Committee (H8016).

### Procedure

All participants undertook a semi-structured, in-depth interview with the lead researcher (MC) (i.e. face-to-face or via Zoom) with a digital audio recording of the session obtained. During the interview, participants indicated their professional experience within elite rugby, and where appropriate, their relevant coaching qualifications. Interviews were 30–60 minutes in duration and consisted of open-ended questions. Questions focussed initially on the coach’s philosophy of PA to gain a better understanding of the participant’s beliefs around the benefits and limitations of PA. Follow-up questions focussed on the practical use of PA, and the analysts contribution, to better understand how coaches engage with PA. Finally, knowledge of PA approaches and the tools used to collect that information was explored to better understand coach’s comprehension of PA (see attached supplementary file). During each interview, follow-up probing questions were asked, based on responses, to gain further insight from participants. The literature in the area of exploring coaches perspectives are in line with the sample size utilised in this study [22,23], however this may also be subsequently influenced by the relatively small population pool of professional rugby coaches, and those who have access to performance analysts.

### Data analysis

All interview recordings were transcribed verbatim with the information thematically analysed [24]. The five-stage process of analysis included: 1) familiarisation of data set; 2) generation of initial codes; 3) identification of themes; 4) review of themes; and 5) final definition and categorisation of themes [24,25]. Two authors (MC, JC) undertook independent reviewing of the transcripts, designed thematic codes, and identified common themes before comparing the data [25,26]. Reflexive thematic analysis was used due to its researcher subjectivity and interpretationof the data to explore coaches ideas and perceptions surrounding performance analytics and its added value to RU [27,28].

In order to ensure trustworthiness of the data, a number of approaches were adopted throughout the preparation, organisation and reporting phases of the study [29]. Throughout the interviewing process, interview audio or transcripts were shared amongst the research group and used to critically reflect on the interview. Additionally, a diary was maintained by the interviewing researcher (MC) to record ideas, questions, and observations from each interview to support the thematic analysis. Follow-up discussions were also conducted with participants when revision or clarification of earlier statements was needed (i.e., member checking). Finally, conformability was addressed through the incorporation of exemplar quotations, and presented in the results section.

## Results and discussion

The aim of this study was to explore elite rugby union coaches philosophies on the contribution of performance analysts within a high-performance sporting environment. Coaches interviewed described performance analysts as an integral position within the high-performance environment. Their role in the organization was described as both highly specialized (e.g., collecting, interpreting, and distributing analytical data), as well as involving generic tasks (e.g., operate or assist with technology), while their purpose was framed around providing support and accountability across the high-performance environment (e.g., coaches and players). Coaches also described the necessary attributes and abilities of a highly effective analyst, which were primarily framed around possessing intimate knowledge of the game, creativity, communication skills, and an ability to manage relationships with key stakeholders. Finally, these dimensions were underpinned by the importance of an interdependent coach-analyst relationship, which subsequently influenced each of the three dimensions. These results also provide insight to both men’s and women’s RU and the growing PA demands and expectations of analysts within both groups, with 60% of the participants have worked within both men’s and women’s rugby. The following sections explore how the coach-analyst relationship shapes each of the three major dimensions. Some of the higher and lower order themes have been discussed together to highlight their co-existence and their need to be intertwined within the analyst’s role, purpose, and attributes. The discussion of some of these themes together provides a greater understanding and importance of all these themes within a high-performance environment. Table 1 depicts the dimensions explored by coaches and the higher and lower order themes which emerged from the data.

**Table 1:** List of lower and higher order themes, and dimensions that emerged from semi-structured interviews with professional (n = 5) rugby union coaches.

Lower Order	Higher Order	Dimensions
Collect metrics & data	Specialist	Role of the analyst In the high-performance sporting environment
Filter and interpret metrics		
Deliver information		
Technology support	Generalist	
Knowledge about the sport		
Video capture		
Coach practices	Accountability	Purpose of the analyst To support stakeholders in a high-performance environment
Player performance		
Match preparation	Support	
Player preparation		
Coach-analyst	Relationship management	Attributes of the analyst To translate data into meaningful information
Coach-player-analyst		
Supporting player-coach		
Verbal	Communication	
Written		
Development	Creativity	
Presentation		

### Role of the analyst

Coaches’ description of the role of an analyst in a high-performance environment was stated both as a specialist, and as a generalist. The specialist role encompassed how data were collected, the filtering process and interpretation of ‘big data’ into relevant information, and the subsequent delivery of this information to key stakeholders. In addition to the specialist role, coaches described analysts as having a generalist role that focused on providing technological support. This assistance ranged from simple use of generic hardware (e.g. laptops and cameras) and software (e.g. Microsoft WORD/EXCEL, etc.), to more specialized technologies, such as operating drones or accessing athlete management systems. Coaches in this study viewed these two overarching roles of a performance analyst as beneficial in improving stakeholder observational skills, analysis and feedback that is achieved through the analyst’s use of data collection, interpretation, delivery of information, knowledge about the game and providing support in other areas such as technology.

#### Specialist

To support coaches, the analyst’s role was described as a specialist in performance analytic information to help inform coaches decision-making. Coaches explained that the vast quantities of data available to them can be overwhelming, and it is the analyst’s role to help manage the flow of information to coaching staff. More specifically, the analyst’s responsibility was to drive the collecting, filtering, and interpretation of data, which is then delivered to coaches as meaningful information. One coach described this process and inferred that performance analysts acted not only as gathers of analytical information, but also as the ‘gatekeepers’ of information:

*“I would say it’s [the data] providing the coaching group with the information that they believe is really important to them. So, there’s probably some generic stats that we all agree are important, that are interesting, that often gets shown to the public on the screen and the rest of it, but I think probably the main part of it [performance analysis] is understanding what that coaching group wants*. *And what’s important stat wise to them, what they look for, what they want to be reporting back to the team. If you’re trying to drive a certain element or game plan, what are the stats around, that can help support their coaching.”*

#### Collect data

During the interview, coaches identified various types of data collection expected of analysts. Examples of these types included video footage, statistical data (e.g., gainline, territory, tackles made/missed etc.) and outcome-based data such as win/loss statistics, which have also been commonly reported in the literature. The data collected were reportedly based around the coaches’ philosophy primarily, and what information and key performance indicators (KPI) coaches believed were critical to team success. Most coaches suggested that this collection process started at the beginning of the season, with data and KPI collected, and metrics continually added or removed based on the team’s performance and key foci throughout the season. One coach discussed the importance of collecting data and various KPI for defining team success:

*“Obviously their ability to collect statistics, and data*, *[and] to be able to link that data to the measurable parts that are important to team success. So that would be at the start of a season, a coach would come up with the trademark identities and key performance indicators that are going to be important for that year.”*

The collection of match/training KPI specific to the organization are then used to describe overall team and individual performance. Laird and Lorimer [30] and Sasaki, Furukawa [31] highlighted a number of KPI used by analysts to provide feedback to coaches and athletes that often led to successful performance outcomes within RU. Similar to what has been previously reported in the literature [20], the evolution of KPI data collected throughout a season can influence a coaches implementation of weekly match tactics, training session focuses and potentially provide a more holistic view of particular aspects of performance. Previous studies have discussed the potential use of contextual factors alongside the collected KPI to provide a greater holistic view [2,32]. Whilst the collection of KPI is undoubtedly beneficial in high performance sport, there are calls for performance analytic data to better represent the dynamics and complexity of match-play, by adopting more contextualized performance analytic information [33].

#### Interpret data

Coaches discussed the analyst’s role in categorizing an immense amount of data collected and then filtering that data for coaches. Most coaches indicated that analysts are needed to interpret data, specifically for use during match review of prior performances, and for upcoming preview of the opposition (e.g. highlighting a team’s strengths and weaknesses during match-specific contexts such as attack or defense). The detailed interpretation of the raw data by the analyst was seen by coaches as a crucial step in the analysis process, as most coaches wanted the analyst to tell them what the numbers meant in a simple and practical sense. Specifically, one coach highlighted the potential of data to provide insights for coaches or draw attention to notions previously unperceived:

*“So*, *let’s say work rate. What’s actually each player is [sic] doing? How fast and how far [they are running]? But then not just leaving it as data, what does that actually mean? That’s where I think the role of the analyst is—to link what the coach wants to measure to the sport science part of that.”*

Previous research has also highlighted that performance analytic data, used during the match review and preview processes, improves coach’s decision-making (e.g. provide coaches with information to make informed decisions around player selection, session planning, match tactics and decisions etc.) and athlete performance [2,6,11,15]. Together these findings, along with the findings of this study, indicate that performance analysts play a critical role in supporting coaches in the high-performance environment. Specifically, by identifying patterns from large data sets, which may not have otherwise been considered by coaches, and that provide a strategic advantage to team performance. More empirical work is needed to investigate how analysts effectively filter-out irrelevant data from the large amount of performance data collected [34].

#### Deliver information [Transforming Data into Meaningful Information]

The final specialist domain identified for analysts involved delivering data interpretations to stakeholders/coaches as meaningful information to influence coaching practices, player preparation, player selection, or behavioral change in players. For example, Carling and colleagues [35] reported how match-related performance analytics were utilized to inform the development and refinement of weekly training programs. Previously, the presentation of relevant match data, in a meaningful way, to coaches was reported to be important for the development of weekly training programs for future success [35]. One coach highlighted the interaction between knowledge of the game and data interpretation as vital for an analyst:

*“I’ve seen other performance analysts who have the experience and ability to say*, *‘okay, here’s the data, but here’s the actual valuable part of that data’, and they can rationalise it down to the most salient, important factors, and then be able to give that to the coach.”*

The importance of delivering *meaningful* information was further expanded upon, with the suggestion that coach-analyst relationships were a vital cog within the specialist domains. That is, in order for performance analysts to be successful at delivering meaningful information to coaching staff, the analyst must have an intimate understanding of how coaches utilise performance analytic information to shape their practices. One coach discussed what impact the delivery of meaningful information had within their environment:

*“I think the real value of an analyst is their ability to bring data*, *usable data, to the floor and make it incredibly public. And again, being able to work with a coach to understand what is [it] that’s going to have the biggest shift in behaviour.”*

Coaches also described the importance of analyst’s delivery methods in effectively conveying the intended information to stakeholders. Verbal presentations and brief written reports were all mentioned by coaches as being effective methods for analysts within their respective programs. Further, this outcome confirmed earlier reports that written reports and presentations were common and effective ways to convey post-match statistics for coaches and to display key opposition preview information to players [2]. One coach highlighted the versatility required of an analyst when presenting data to the coaches:

*“And then [performance analysts] being able to present them through different mediums, whether that be PowerPoint, Keynote, and all those other flash things that you do. Then also, there’s huge amounts [of] scouting, and just being able to really present that information simply and accessibly so the coach can do stuff with it.”*

Alongside the mode of delivery, the *timing* of analyst’s delivery was also another important factor identified by coaches. While delivering information for match preview and opposition review afforded analysts time to prepare, coaches also described situations where analysts were expected to deliver immediate feedback to either coaches or players during practice. By doing so, coaches noted the improved delivery of their own feedback to players, thanks largely to the analyst’s ability to strengthen coaches’ feedback with supportive video footage or notational analysis. The use of immediate feedback through analyst-coach collaborations within training sessions and matches has been shown to improve real-time team performance [36–38] and highlights the value of a good alliance between analysts and coaches. One coach highlighted the utility of feedback within the training environment and the role the analyst played in achieving that, summarizing the concept of all coaches interviewed in the current study:

*“So, when I have a drone connected to footage so we can film an attack shape…, on the radio, you say, ‘Mate, can you clip that last two phases?’ Team runs over, and then the coach will be down [to meet the team], the coach will talk about it and back on field. So, you’re getting automatic feedback loop, live. So that’s been a massive improvement for us, and it’s what most teams should really be pushing towards or are doing.”*

#### Generalist

Although analysts were highly regarded by coaches for their specialist role, another critical element to the role of an analyst, was their ‘generalist’ abilities including knowledge about RU and technology. Performance analysts having a broad, generalist skill set not only provided great assistance to the coaching staff, but also to the players and support staff within the organization.

#### Knowledge about the game

*Coaches commonly highlighted that an analyst* should have an extensive knowledge of RU, including the laws of the game, technical skills, and physical demands required of the players. Specifically, a knowledgeable analyst was described in terms of their ability to anticipate what information and KPI would best serve coaches during match preview and review processes. This finding is in line with previous research that highlighted the importance of a skilled and game-knowledgeable analyst who could identify KPI, from the vast amount of data available, to support coaches and team performance *[30,31]*. While all coaches highlighted this as a crucial trait in effective analysts, one coach went on to further explain that knowledge of the game needs to be supported by a proactive work ethic:

*“If you get somebody [analyst] who shows a hell of a lot of initiative and is really good at their analysis and they understand the game then they are in a position where they can actually be really proactive and providing you information around that review process or the preview process.”*

#### Technology guru

Coaches further described the analyst’s role as a ‘technology specialist’. Coaches stated that analysts were not only required to have an extensive knowledge about the game, but also a broad knowledge of technology. This ranged from knowing how to operate their own analytical technology (e.g., video recording devices, software etc.), but also a general understanding of various forms of technology used within the organization (e.g., computer software including Microsoft Office, projector units, etc.). Coaches described an analyst’s role as being a ‘problem solver’ and being the ‘go-to’ person when it comes to any issues with technology, exemplified by the following quote from one coach:

*“I think in rugby union, probably because we’re not a very well-resourced sport, they’ve [the analyst] almost got to become—and I’ve seen this in a number of different environments—where they are the IT specialist. They are the go-to for everyone that has to do with a computer, unfortunately. Obviously, they’re experienced with a whole raft of things… . Rugby [analysts] are generalists in a lot of things.”*

### Purpose of the analyst

Coaches described the importance of the analyst’s relationship between their practical role and their overall purpose within a high-performance unit. According to coaches, an analyst’s primary purpose within the team environment was to provide accountability and support for coaches and players (i.e., key stakeholders) with the use of objective data. While Wright, Atkins [20] outlined the role of an analyst there is little information about why their role is important in professional sports. Therefore, to our knowledge, the current study is one of the first to explore the purpose of performance analysts in a high-performance sporting environment.

#### Provide accountability & support

Coaches discussed the importance of the analyst providing evidence of accountability for both coaches and players, as well as overall team performance. The collection, interpretation and delivery of information collected by a PA provided conclusive evidence to support the accountability of stakeholders, such as player’s match performance and training efforts, or coaching practices and selection processes. To date, there have been very few studies which have specifically discussed how PA data were used to hold both coaches and players accountable for their performances. It is worth noting however, that a coach’s reliance on performance analytics, to hold them and their team performance accountable, suggests these coaches likely possess a growth mindset towards self-improvement. One of the coaches interviewed, suggested the provision of accountability within the data collected and presented was paramount to the analyst’s role within their program:

*“I think that’s probably, for me, one of the most important areas, around are we measuring the thing [KPI] that we said we wanted to be the best at, and then are we holding our team accountable for their performance, and are our measures reliable and accurate? And then how we’re displaying that publicly. I see that as the role of the performance analyst. Probably, for me, the number one thing. Measure it, make it public, make the players accountable for that.”*

Another purpose of analysts in the RU high-performance environment, as described by coaches, was the integral support they provided to the overall performance of the team. Examples of this support included assisting coaches in developing match tactics, preparation, and upskilling coaches, as well as reinforcing player development in areas such as skill execution, role clarity, game intelligence, confidence and motivation.

#### Coaching practices

The information provided by the analyst was suggested to support coaches decision-making processes, such as coaching practices and player selection. Coaches described the benefit of metrics and analyst input to support and justify training plans, match tactics, and provide confirmatory information to other stakeholders. One coach provided a drill specific example whereby using data to support their instructions, or within-drill cues, enhanced their own coaching efficacy:

*“I think it’s big because I think in a lot of coaching, we can put out a broad statement like ‘Girls I want a lot more from you this week’. And any coach could say that, whereas I think an awesome coach can say ‘Okay I want more effort, and here’s how we’re going to track it’. So, like if that’s a 3-metre acceleration and this is how we are going to track it.”*

With regards to player selection, coaches described the importance of being provided with objective data, to support player selection. Coaches described the influence that PA data can have on their player selection for upcoming matches. The PA data provides coaches with factual information that can support their decisions when selecting players for specific positions. Previous work in badminton and RU has also reported PA data usage to support coaches practices and player selection [18,39]. One coach interviewed discussed how the factual information provided by the analyst can provide further insight into the perceived performance of an athlete:

*“So, on the run and also after the game, there’s a whole lot of data, but it’s being able to strip it down to what’s critical for your team to be successful between Sunday and Saturday, and sometimes you’ll get a surprise. So, you’ll watch the game, and you think you see [something], ‘Oh, I don’t know whether he played that well’, and you watch the game again, [and] you go, ‘Oh, he wasn’t as bad’, and then the analyst will go, ‘Oh, he actually had 13 carries and they all got over the gain line’.” So, you kind of back each other up.”*

#### Player performance

Coaches highlighted that the analysts also provided players with objective data on their overall performance. This process allows both stakeholders to identify player’s strengths and weaknesses, and develop tactics to improve skill execution or game knowledge for improved overall performance. Francis and Jones [36] reported that players believed that the use of objective PA data held them accountable for their performance and enhanced their game tactic understanding. Therefore, the ability to provide objective data can be beneficial in shifting athlete behaviors and holding them accountable for these behaviors. One coach highlighted the overall impact that analysts can have on player performance:

*“I think that’s the real skill, in that you can shift behaviour through evidence of data that supports what the coach is saying. So, I think sometimes as coaches we make that stuff up. But if we’ve got actual data that supports that, then it makes the case incredibly compelling. No one likes to see their name in red, and you’re below the line, that shifts behaviour, or can do. The ability of the analyst to highlight that stuff [metric/KPI] and bring it to the forefront, that will shift a team and move it.”*

#### Match preparation

Coaches highlighted that analysts were key to identifying the oppositions strengths and weaknesses. This allowed coaches to formulate their own match tactics to target areas of the opposition’s game and that would benefit their own team’s performance. Based on a coach’s philosophy, a coach will often focus on either the oppositions strengths and weaknesses, their own teams strengths and weaknesses or a combination of both. Fernandez-Echeverria, Mesquita [6] and Middlemas, Croft [15] also reported that coaches in volleyball and RU valued the analysis of opposition performance data as it influenced coaching practice and development of team tactics to increase the chances of success. Fernandez-Echeverria, Mesquita [6] discussed the use of opposition analysis during the preparation phase of a coach’s weekly cycle. Analysing opposition performance can provide useful information that can allow a team to best prepare for a match by developing defensive strategies and attacking plays to exploit the oppositions weaknesses. One coach emphasized the importance of PA, and indirectly an analyst, in match preparation:

*“I think the beauty of analysis is finding out how teams struggle, how do they lose. Tell me that, because that’s what you’re ultimately trying to do, you’re trying to beat them. So how do we go about beating them. There’s lots of different theories regarding strengths [sic] approach. Where can we exploit? Where are opportunities? Get that information. That’s very valuable.”*

Analysts were highlighted by coaches as crucial to their own upskilling of practices and knowledge surrounding the game. For example, educating new coaches on how information provided by the analyst could assist coaches in developing new match tactics (e.g. source of possession and average number of phases it takes for the opposition to score) or training plans to improve athletic performance (e.g. metres per minute for particular drills and how that relates to physical matches demands). One coach highlighted the benefits of an analyst and PA to upskill coaches:

*“I think also, there’s a big part of it where the analyst can actually upskill the coach, and the coach obviously has to be open to it. It’s all relatively new to a lot of new and upcoming coaches, especially [sic] players. I was lucky throughout my career where I was constantly being giving stats. I found with some coaches that are [sic] come through amateur clubs, club systems or school systems that has [sic] never seen a stat. So, they don’t know what’s important, or what could be helpful. So, I think there’s definitely that element there which is important.”*

The ability of the analyst to upskill coaches around PA data and practices has not been reported previously to our knowledge and therefore highlights just another unique aspect that an analyst adds to a high-performance setting.

#### Player preparation

Analysts were also described as critical for the provision of objective player performance feedback. Specifically, when coaches discussed with players about performance-related behaviors such as role clarity, skill execution, and game intelligence. This additional layer of support was further described in terms of providing additional indirect benefits according to coaches, via improving player confidence and increasing motivation. Francis and Jones [36] reported similar findings, where players were found to improve their tactical understanding, motivation, and confidence when included in the PA process. One coach elaborated on the implications of an analyst providing key information on a player’s confidence levels:

*“It [providing key information] could also be as a motivating factor for a particular player because you could actually have a player who may not recognise their value to the team, however you can say well look at your numbers. ‘The numbers don’t lie here; I’ve got footage I can show you. Look at the numbers. You’re doing that regularly throughout games. You know you’re carrying 25 metres every time you carry’–like, that’s brilliant from a player confidence point of view.”*

### Analyst attributes

The final dimension identified for an analyst within the elite RU environment was a range of personal attributes. Relationship management, effective communication and creativity were prominent thematic attributes identified by the coaches when describing a valuable analyst.

#### Coach-analyst relationship

The successful relationship between the analyst and key stakeholders (i.e. head coach) was labelled as crucial for an effective analyst in the current study with trust, communication, acceptance, respect and mutual goals important for this relationship. Studies across multiple sports including, rugby league, basketball, hockey, football and RU have reported the importance of developing a strong and collaborative relationship between a coach and their analyst [2,20,40]. It is suggested that a strong and collaborative relationship will allow coaches and analysts to openly discuss various ways to achieve successful team performance. Bateman and Jones [21] highlighted trust and honesty as the most highly rated attributes of analysts by coaches. The coaches interviewed discussed the importance of trust within the coach-analyst relationship. If a coach cannot trust in their analyst’s ability to perform tasks required of them to a high standard this can cause tension in their relationship and can ultimately impact the entire teams performance. This mistrust formed between the coach and analyst could lead to the PA data provided being used ineffectively or inappropriately. All coaches described the importance of a positive coach-analyst relationship as demonstrated by the following from two of the coaches:

*“So, my view is that performance analysts should be proactive in their approach with the coach, and it’s still the coaches prerogative to say, ‘okay, that’s not quite what I want’, or to give them direction. But I would be looking for a real proactiveness from them.”*

Coaches identified an analysts’ enthusiasm to be fully involved within the coaching group as important. For example, regular engagement with the coaching group could lead to greater communication including robust discussion of relevant data and methods, further strengthening this important staff relationship.

*“But I think for me, the more a performance analyst can be proactive. So, they’re really guiding and doing the thinking for the coach. Pre-empting, using their initiative, really thinking about, ‘okay, what can I do to really add value and provide input.’ Thinking ahead, knowing there’s a meeting coming up, and yeah, just working well and doing their role really well.”*

The ability to maximise resources and time efficiency, such as successfully designing and implementing specific KPI that align with a coach’s philosophy, coaching practices and match tactics, further highlights why the coach-analyst relationship is so critical to team success [20]. One coach explained the utility of including the analyst within the discussions and meetings amongst coaches, within the team and around the facility for establishing best practice:

*“I think that’s the analyst being in the room, being involved with coaches and team meetings. I think it’s probably easy to go sit in the back room and get through your work, but maybe it’s better time spent being in a team meeting, understanding what’s being spoken about. And how good is it if the next day if [sic] they [the analyst] say ‘I heard in the team meeting that we are looking for this. Here’s how we trained and here’s how they actually performed on what you [the coach] talked about’.”*

However, a critical element of the coach-analyst relationship was how well the analyst understood what the coaches wanted from them. For example, anticipating coaches’ performance data needs, filtering key information from ‘big-data’ sets, and delivering meaningful information to coaches, based on each individual coach’s philosophy and coaching style. An analyst’s ability to anticipate what information their coaches would benefit from, to support their coaching practices, was rated as critical by coaches [21]. An example that epitomised all coaches responses is presented below:

*“The analyst is a part of your coaching team, so, you’ve got to be able to [sic]. I need to know what the analyst can do, and I need to know how the analyst works and I need to know how they measure everything. And then he [the analyst] needs to know how I coach and what’s important to me and what type of rugby you want to play so that he can then highlight things that I think are relevant.”*

#### Player-analyst relationship

Interestingly, the coaches rated the player-analyst relationship to be of little importance in the high-performance environment. Coaches expressed their concern that analysts providing large amounts of information to players may cause an ‘information overload’. Coaches believed this overload may result in players overthinking situations, and loss of trust in the coach that compromised the coach-player relationship. Bampouras, Cronin [17] reported that the player-analyst relationship was limited at the elite level with coaches acting as ‘gatekeepers’ of information and limiting the development of an effective player-analyst relationship. It should be noted that athletes reported feeling ‘alienated’ during the PA process when coaches acted as the ‘gatekeeper’ of information, thereby straining the player-coach relationship and providing a further barrier to successful team performance [17]. One coached described this viewpoint from the perspective of overloading players with information becoming detrimental to their performance:

*“So, we’ve got a lot of information, and you can’t give too much to a player because it’ll throw them. And you don’t want them thinking—any thinking slows them down. So, you’ve got to work out firstly what’s really critical. There can be some information where you go, ‘You know what? It’s not relevant. It doesn’t matter. We don’t need to tell them that. We know that. They don’t need to know that.’ So, it’s working out as coaches and analysts, if the analysts gave the players all the information, you’d fry them. They’d be the slowest moving humans because they think too much.”*

Instead, analysts were described as necessary to help support coach-player relationships, specifically when discussions centered around optimizing team training or performance. For example, an analyst could provide video footage (immediate or delayed) to a player and coach that shows that coaches were providing players with objective rather than subjective feedback [36]. This confirmation by an analyst created a greater level of trust between players and the coaching group and a stronger relationship between coaches, analysts and players [36]. Similarly, one coach in the current study provided an example of how data could help identify performance fluctuations in the training environment:

*“So, if [the analyst] is coming to me with ‘Here are the stats, here are the people that have been really poor this week on training’ and I’m seeing really bad trends and they might say these five people might be worth having a conversation with. So, that sort of information is really valuable and quick and easy rather than me having to work that out of the stats, a quick comment from [the analyst] resolves that really quickly.”*

#### Communication

The ability of an analyst to communicate, in written and verbal ways, to stakeholders was identified by coaches as another important attribute and confirms earlier reports [2,20]. Previously, coaches were reported to use several visual modalities (e.g. video footage, animations, graphs etc.) created by the analyst. The use of various types of media to communicate important information to the playing group allows the delivery of accurate and detailed information to the player [41–44]. The ability of the analyst to converse with their coaches effectively allows for a clearer understanding of coaching philosophies and match tactics to be obtained. For example, Wright, Atkins [20] highlighted the necessity of effective communication between analysts and coaches, to ensure the KPI collected, interpreted and delivered were those that reflected the coaches philosophies and match tactics. One coach explained how he believed the communication of data may be best presented graphically rather than numerically emphasizing versatility in communication skills as a necessary requirement for performance analysts:

*“I think how you present your numbers has a greater impact on the numbers themselves, because if you just give them the numbers themselves the players don’t get it and they find it very hard to contextualise it. That might just be presented on a speed dial or circular graph or pie graph or something but if it resonates with them, they go ‘ah I get that’. And then you’re able to better put it into the context of the game and then they have a behavioural change to then create effect.”*

#### Creativity

Finally, coaches described an analyst’s ability to develop new and innovative ways to collect, analyse and deliver information, based on large amounts of data available, simply and meaningfully as important. An analyst’s ability to identify new ways to collect or interpret the data available to them could allow them to potentially come up with a better way of analysing athletic performance (e.g. a specific combination of KPI to provide a more holistic view of performance taking into account contextual factors). Being able to develop creative and interesting ways to deliver the important information was suggested by coaches to engage the players more than just presenting number/s on a screen. The analyst’s ability to understand and be creative with ‘big data’ was also described by coaches as potentially providing teams with a ‘competitive edge’. For example, filtering through the large amounts of data available and finding the one or two KPI that can be used in a new way to provide their team with a ‘competitive advantage’. One coach provided an example of a situation where the analyst created a new metric that could potentially provide that team with a competitive edge over their opposition if executed:

*“OK yeah like he sort of come back and said ‘are we going to use that metric? Is that going to be effective when we present it to the players?’ And it was a case of ‘Yeah that one’s not, but this one is.’ One of the metrics was around airtime and distance on our box kicks. So, we met, and we measured airtime and, we measured the distance and, then we correlated that with the speed of our outside back like our chasers. And so, we pretty much work out that [X distance] on two out of three of our nines [scrum half] was the optimum distance on a box kick so every time we trained a box kick it was [X distance].”*

## Conclusion

Based upon the perceptions of elite Australian RU coaches, the current study sought to explore and understand coach’s philosophies surrounding PA within RU and its implementation within their programs. From this, coaches described the role, purpose, and attributes of a performance analyst within their elite RU environment. Perhaps most importantly, it was found that coaches philosophies and the coach-analyst relationship was critical for the analyst to fulfil the roles and purpose of their position in a high-performance environment. For example, while the specialist role of the analyst entailed collecting metrics, filtering it into meaningful information, and then delivering that information to coaches, the selection of metrics and method of delivery was heavily influenced by the coach-analyst relationship. These findings suggest that the performance metrics used by professional clubs are largely dictated by the coaches needs and analysts’ ability. Overall, the coach-analyst relationship was perceived as a key element within a high-performance environment, as it underpinned the analyst’s purpose and contribution to team success. For example, coaches discussed not only the role of the analyst, but how the information provided supports coaches and players during match preparation and provides accountability to player performance and coaching practices. This indicated that analysts had a greater purpose within a high-performance setting, adding multiple layers to their overall role. These data supports the need to professionalise the role of an analyst not only in RU but across all sports with the majority of analysts within the industry working long hours and holding both tertiary and coaching qualifications within their sports. Finally, individual characteristics such as effective relationship management, communication and creativity were highlighted as important attributes of an analyst which add benefit to a high-performance environment. These attributes are important for building relationships and providing teams with the creativity to explore new ways to gain a competitive edge over the opposition. This area would benefit from future work investigating the philosophies of performance analysts, including how they effectively filter-out irrelevant data from the large amounts of PA collected.

## Supporting information

S1 File (DOCX)
