# Supplementary material for: Exploring rugby coaches perception and implementation of performance analytics
Source: PLoS One. 2023 Jan 24;18(1):e0280799. doi: 10.1371/journal.pone.0280799 (PMC9873156; doi:10.1371/journal.pone.0280799)
Supplement: S1 File — (DOCX) [file pone.0280799.s001.docx]

## **Supplementary File:**

Planned Interview Questions

1. What do you think a performance analysts’ major role?

- What impact do you think this has on coaches and athletes?

1. Do you find performance analysis useful within rugby union?

- If so where do you find its use is most beneficial?

1. How important would you say performance analysis in your coaching philosophy?

- Why do you think that is?
- Where do you find its use most beneficial?
- What impact do you think this has on coaches and athletes?

1. How important do you think the relationship between a coach and the performance analyst is?

- Why do you think it is important?
- How does a performance analyst understanding of the coach’s expectations and requirements benefit you and the team do you think?

1. When preparing your team for their upcoming match how do you find performance analysis useful in effectively delivering your intended message to the playing group?

- How would you use it?
- What would you find beneficial?
- What do you find isn’t as important?

1. Can you describe for me what happens during the week leading into a game?

- What part does performance analysis play here?

1. Who is responsible for putting together this information?
2. Does all this information get shared with players? Or is some of the information filtered down?

- Who decides what gets shared and who is responsible for sharing that information?

1. Do you find performance analysis is a useful when looking at the upcoming opposition?

- How would you use it?
- What do you find beneficial about it?

1. What do you think isn’t important when it comes to looking at the opposition?
2. Can you describe to me what happens after a game?

- What part does performance analysis play here?

1. What do you talk about during player reviews?
2. Where does the information come from during these player reviews?

- Is there someone that is responsible for collecting and playing video clips or sharing the data with everyone?
- Are you using the performance analysis data to drive what you are saying to the players? Or are you using it more to support what you saw in the game and back up what you are saying?

Who decides what information is shared during these players’ reviews?

- Are there other coaches involved? In what capacity?

1. Is training during the week pre-planned/ to what extent?

- To what extent do you have your in-season training planned?
- How flexible or adaptable is your weekly training session?

1. To what extent does prior match performance inform training for the following week?

- Does this also apply for upcoming opposition?

1. Does this information come from your observations of the game or from performance analysis data?
2. What factors influence your decision to select or drop a player for an upcoming game?

- What is an example where performance analytics influenced your selection or non-selection of a player?

1. How common is it for you to utilise performance analytics to inform your selection practices?
2. How do you think is the best way to implement performance analysis effectively within your team?
